# Squamous Cell Bladder Cancer: A Rare Histological Variant with a Demand for Modern Cancer Therapeutics

**DOI:** 10.3390/cancers17020169

**Published:** 2025-01-07

**Authors:** Scott D. Bell, Anthony E. Quinn, Alfred Bajo, Trenton G. Mayberry, Braydon C. Cowan, Austin J. Marrah, Mark R. Wakefield, Yujiang Fang

**Affiliations:** 1 Department of Microbiology, Immunology & Pathology, Des Moines University, West Des Moines, IA 50266, USA; scott.d.bell@dmu.edu (S.D.B.); anthony.e.quinn@dmu.edu (A.E.Q.); alfred.bajo@dmu.edu (A.B.); 2 Department of Surgery, University of Missouri School of Medicine, Columbia, MO 65212, USA; tgm9h3@health.missouri.edu (T.G.M.); bcy9y@missouri.edu (B.C.C.); ajm4cc@missouri.edu (A.J.M.); wakefieldmr@health.missouri.edu (M.R.W.); 3 Ellis Fischel Cancer Center, University of Missouri School of Medicine, Columbia, MO 65212, USA

**Keywords:** squamous cell carcinoma, bladder cancer treatment, bladder cancer epidemiology

## Abstract

Bladder cancer is among the most common form of cancer worldwide and is predicted to increase in incidence and mortality over the next decade. Squamous cell carcinoma of the bladder is a rare histological variant typically associated with schistosomiasis, also known as bilharzia, a parasitic infection caused by flatworms called schistosomes or blood flukes, and is generally seen in underdeveloped nations. However, squamous cell carcinoma of the bladder still represents nearly 5% of bladder cancer diagnoses in the western world. Transitional cell carcinoma is the predominant histological variant of bladder cancer found throughout the western world, and nearly all disease indicators and treatments for bladder cancer are driven by this common variant. Squamous cell carcinoma of the bladder shows characteristic features that differ from transitional cell carcinoma, such as differing levels of protein indicators and different response rates to traditional bladder cancer therapies. Common treatment methods for squamous cell carcinoma of the bladder include radical cystectomy, chemotherapies, and radiation. Reviewing the previous literature on the management of squamous cell carcinoma of the bladder, it becomes apparent that this variant needs to be treated differently than common bladder cancer variants and a proper management course needs to be set in place to maximize positive patient outcomes. Such a study will be very helpful for urologists and oncologists to manage patients with bladder squamous cell carcinoma.

## Simple Summary

Bladder cancer is predicted to have an overall increase in incidence and mortality in the next decade. By far, the most common histological type of bladder cancer is transitional cell carcinoma, which is the focus of most of the research and clinical trials being performed to control bladder cancer incidence and mortality rates. A less common subtype of bladder cancer, squamous cell carcinoma, shows characteristic disease progression and etiology when compared to transitional cell carcinoma. Squamous cell carcinoma of the bladder is commonly associated with schistosomiasis in developing nations and recurrent urinary tract infections and indwelling catheterization in the western world. Due to its unique characteristics, more research is needed to properly treat squamous cell carcinoma of the bladder with modern therapeutics.

## 1. Introduction

Bladder cancer (BC) is the ninth most common cancer type in the world with over 600,000 cases reported in 2022 [1]. Bladder cancer caused 213,000 deaths in 2020 and was found to have both incidence and mortality rates nearly four times greater in men than women [2]. BC is the fourth most common and eighth most deadly cancer in the US for men. By 2024, it is estimated that the annual number of BC cases and deaths will increase to 991,000 and 397,000, respectively [2]. The most substantial risk factors for BC are tobacco smoking, occupational exposures to aromatic amines or poly-cyclin aromatic hydrocarbons, and various dietary factors [3]. 

Transitional cell carcinoma (TCC) is the most common histological variant of BC, with 75% of BCs having pure TCC histology [4]. Squamous cell carcinoma (SCC) is the second most common histological variant [5]. SCC typically evolves from the TCC variant and commonly presents with features of TCC, thus the diagnosis of solely SCC of the bladder is a rare occurrence. SCC BC is usually found in its invasive stages, often with keratinization present (Table 1) [6,7,8]. Locoregional recurrence is the main cause of death after initial treatment [9]. Further, SCC BC is shown to have less incidence of metastases to the pelvic lymph nodes and to distant tissues when compared to TCC BC [10]. 

Bilharziasis, also known as schistosomiasis, is an ongoing and unceasing issue in developing nations [11,12,13]. Schistosomiasis is an infectious disease normally caused by parasitic flatworms, called schistosomes or blood flukes, that live in freshwater. Commonly, these flatworms penetrate the skin or mucous membranes and will then migrate throughout the urothelial tract to the bladder, where they can cause constant bladder irritation. Schistosomiasis is most common in tropical regions, and specifically tropical areas with poor sanitation practices. Most of the cases of schistosomiasis in the world occur in sub-Saharan Africa and nations such as Nigeria, Tanzania, Mozambique, Sudan, and the Democratic Republic of the Congo. Areas of Northern African and the Middle East also harbor schistosomiasis in nations such as Egypt, Yemen, and Sudan.

**Figure 1:**
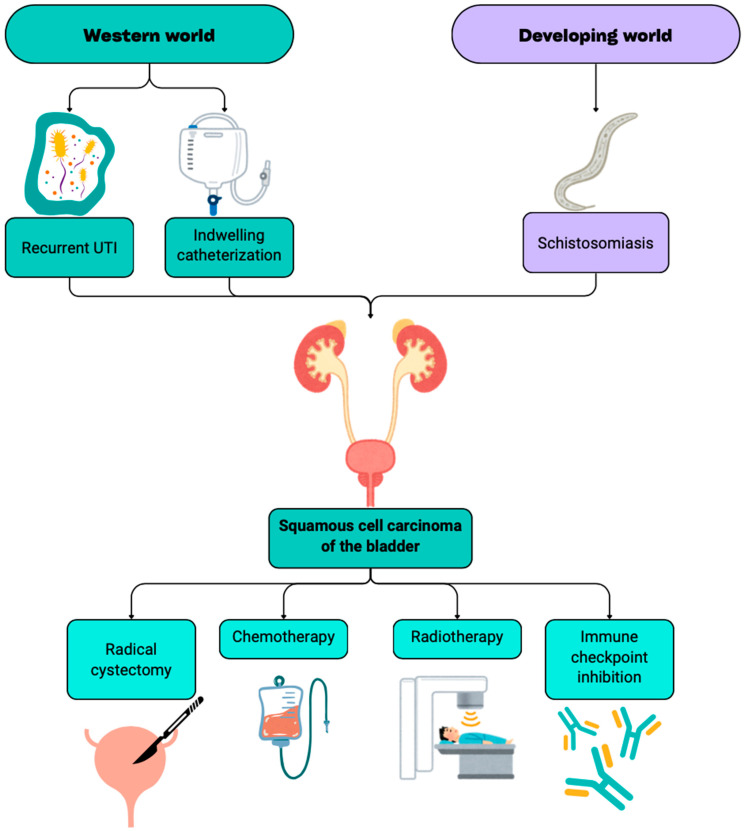
The common etiology and treatment options for squamous cell carcinoma of the bladder.

SCC develops from a process referred to as squamous metaplasia, where transitional cells of the bladder surface will undergo constant irritation and differentiate to squamous cells. BC associated with schistosomiasis is commonly found in the newly formed squamous tissue of the bladder and is usually the causative agent for the SCC BC presentations seen in developing nations (Figure 1) [14,15,16]. The increased incidence of SCC BC in developing nations is suspected to be due to their lack of control of schistosomiasis infections, though there are ongoing efforts to decrease the disease incidence [17]. Besides being a principal cause of SCC BC, schistosomiasis has been shown to cause contracture and ulceration of the bladder along with the ureteral stricture [18,19,20].

Egypt presents some of the highest incidence rates of schistosome related BC. A study focusing on Egyptian patients with bladder carcinoma found that 82.4% of patients presented with a concurrent schistosome egg species present [21]. Of those patients who had egg-positive specimens, the predominant variant of BC was SCC of the bladder [21]. Another study focused on Egyptian cases found that 105 of 148 patients, or 70.9%, that received cystectomy for BC showed bilharzial pathology and 51 of 148 patients, or 34.5%, showed SCC BC pathology [22]. Also, Africa presents high rates of SCC BC with an estimated 53–69% of BC cases that are of predominate SCC histology [16]. Areas of Africa that are home to the highest rates of schistosomiases are areas of poverty and tropical regions. Further, most of the SCC BC cases in these areas are also caused by schistosomiases disease. In South Africa, school-aged children are most at risk for developing schistosomiases [23]; thus, there is a rationale behind the major push in disease knowledge targeted at the younger generations throughout Africa. 

Recently, there has been a major push for better control of bilharziasis in Egypt, with a correlating decrease in the incidence of BC. Over a 37-year period beginning in 1970 and ending in 2007, Bilharzia infection has dropped from 82.4% to 55.3%. Also, in these areas there has been a significant decrease in SCC histology that has followed, from 75.9% to 28.4% when compared to the incidence of other BC histological variants [24]. Freshwater snails play a crucial role in the lifecycle of schistosomes, as they are the intermediate host of the parasite [25]. In the 1930s, workers drained Egyptian river-fed canals to collect the intermediate snails [26]. Praziquantel, an anti-parasitic used to treat a number of parasitic worm infections, is commonly administered in the wake of endemic schistosomiases infections [27]. Today, combining snail control along with preventative mass drug administration is thought to be the most effective means of schistosomiases control [28]. Additionally, government-backed campaigns throughout 1960–1980 promoted the widespread use of anti-parasitic medications and advised farmers not to urinate in the Nile since it would release the parasite, restarting the life cycle [29]. While significant efforts have been made to reduce the prevalence of parasitic disease, pockets of infection still exist and require further preventative strategies.

Africa is also making strides in the control of schistosomiases. With the decreased cost of praziquantel throughout Africa owed to efforts from global health organizations, endemic areas of sub-Saharan Africa are showing increased use of the drug. Also, pregnant women in these areas have been given approval to use praziquantel [30]. The World Health Organization has drawn attention to increasing the knowledge of schistosomiases in endemic areas [31]. Efforts have included spreading knowledge of the use of praziquantel, mass drug administration programs, and signs and symptoms of schistosomiases [32].

The standard methods of examining and staging typical BC such as cystoscopy, transurethral resection of the bladder tumor (TURBT), and imaging still apply to SCC BC, though detection and staging of this variant are crucial as the percentage of variant histology correlates to the effectiveness of chemotherapy [33]. To detect this specific variant of BC, various techniques must be performed as accurately as possible. Previous studies have shown that TURBT can be relatively inaccurate at detecting variant BC histology such as SCC [34].

SCC BC does show characteristic biomarkers that may allow for enhanced detection in the future. Psoriasin is a calcium binding protein expressed by squamous epithelial cells that has shown upregulation in SCC BC pathology. Psoriasin is excreted in the urine and may be a valuable biomarker that clinicians can use to characterize BC variant histology [35]. Badr et al. found that over 73% of muscle-invasive SCC BC cases they examined presented with heavily expressed p53; however, p53 mutations are not of value when grading or staging the disease [36]. Further, upregulation of p53 oncoprotein has been shown in both schistosomiasis-associated TCC and SCC BC [37]. Also, it has been shown that bcl-2, HER/neu, and MIN-1 expression provide little value in grading or staging the disease [36]. 

In the Western world, less than 5% of BC diagnoses are of squamous histology [6,38,39]. These rare occurrences of SCC are usually attributed to prolonged indwelling catheterization use, recurrent UTI, or spinal cord injuries [40,41,42]. The incidence of SCC BC in the western world is increasingly rare and is commonly not associated with underlying bilharzial pathology [10,39]. Like typical BC presentations, smoking remains a predominant risk factor in the development of SCC BC [43]. Notably the incidence and mortality rates of BC are decreasing in the western world and increasing in developing nations [44,45]. This may be due to better utilization of disease prevention measures in the developed world as opposed to developing nations that historically lack strong infrastructure to support disease prevention measures, treatment, and knowledge. 

## 2. Radical Cystectomy

The current gold-standard treatment for SCC BC is radical cystectomy (RC) with lymph node dissection [7,9]. During RC, the entire bladder and surrounding pelvic lymph nodes are typically removed to decrease the systemic spread of cancer. RC today is performed in open, laparoscopic, and robot-assisted settings. Open and robot-assisted RC show similar post-surgical oncologic and safety outcomes [46]. Laparoscopic RC shows a decreased distant metastasis rate compared to open RC for BC and is becoming the preferred modern method of operation [47]. During RC, lymph node dissection can be performed where lymph nodes that show cancer metastases and possibility of cancer metastases can be removed to offer better survival benefits [7]. RC has been shown to be the preferred method for surgical treatment of SCC due to an increased cancer specific survival (CSS) rate compared to other treatment modalities [7,48]. Further, in a retrospective SEER database study, it was found that for patients with non-metastatic SCC of the bladder, RC showed superior overall survival (OS) when compared to radiotherapy (RT) [49].

Due to SCC usually presenting late in progression and in invasive stages such as T2-T4, RC becomes the practical treatment method [38,50]. For SCC that has progressed to muscle-invasive bladder cancer (MIBC), RC is widely considered the standard due to its oncologic success [51]. If the SCC has progressed to MIBC, RC is indicated at the earliest available time to increase chance of survival and decrease distant metastases [52,53,54]. 

RC can be combined with neoadjuvant therapeutic techniques to increase patient clinical benefit, though results have been confounding. Traditional neoadjuvant therapies preceding RC are chemotherapeutic drug delivery and targeted radiotherapy (RT), though RT is less common in current practice. It has been shown that patients with SCC show decreased CSS and worse mortality rates compared to those with the more common BC variant when treated with neoadjuvant platinum-based chemotherapeutics [55]. Similarly, patients treated with a neoadjuvant gemcitabine and cisplatin combination chemotherapy were found to have no improvement in OS compared to RC alone [56]. Further, in a National Cancer Database query, Matulay et al. found there is no OS benefit for neoadjuvant chemotherapy delivery preceding RC [57]. Though, it has been reported that neoadjuvant chemotherapy is advisable when the primary tumor has less than 50% squamous cell variation in primary cancer histology to increase OS [33].

## 3. Chemotherapy

Because of the rarity of SCC of the bladder as compared to urothelial carcinoma (UC), also known as TCC, many of the chemotherapy regimens for SCC mimic the treatments that UC presentations receive. Interestingly though, SCC variants have been shown to have different response rates to chemotherapy than UC variants where SCC is typically less responsive to chemotherapeutics [39,58,59]. Typically, SCC presents as a chemotherapy-drug-resistant form of BC that has a less desirable response to neoadjuvant and generally administered chemotherapeutics [13].

Chemotherapeutic agents are commonly administered as neoadjuvant therapies to RC in many circumstances as standard practice to elicit a positive clinical oncologic response (Table 2). To treat SCC variants, typical BC chemotherapeutics such as cisplatin, carboplatin, and paclitaxel have been evaluated in clinical settings. Cisplatin has been a long-standing drug of choice in the treatment of invasive UC bladder cancer [60]. But in patients with UC with squamous differentiation BC variants, neoadjuvant cisplatin has been shown to have no clinical benefit compared to receiving no neoadjuvant therapy [61]. Similarly, it has been shown that in patients with primary SCC of the bladder, hospital stay length and OS were not statistically different when patients were treated with neoadjuvant cisplatin [62]. Further, for patients with mixed histology tumors with squamous differentiation, carboplatin-based therapies have been shown to provide no increase in OS [63]. Importantly, it has been shown that the effectiveness of neoadjuvant chemotherapies relies on the percentage of squamous variation that has occurred within the diseased bladder tissues [33]. The percentage of squamous variation is important to the extent that patients with <50% squamous variation were 26.7 times more likely to have a pathologic complete response to neoadjuvant chemotherapy versus patients with ≥50% squamous variation being 8.7 times more likely to have pathologic complete response to neoadjuvant chemotherapy [33]. These results highlight the importance of detecting BC with SCC histology as early as possible in disease progression, before the greater squamous variation can occur.

Other chemotherapeutics have varying effectiveness in various retrospective studies and case reports. Miyata et al. used a combination of gemcitabine and paclitaxel to treat patients with a recurrent SCC tumor mass. The patient’s tumor showed a marked decrease in pelvic tumor mass volume, nearly decreasing in size by half, but the tumor eventually persisted, and the patient eventually died from the tumor [64]. Galsky et al. used a combination therapy of ifosfamide, paclitaxel, and cisplatin to treat patients with SCC of the urothelial tract. This combination therapy was administered to a patient with confirmed metastases to the pelvic lymph nodes and the patient was later determined to be disease free after treatment and lived another 8.5 years until death [58]. Further, Hussain et al. tested a combination of paclitaxel, carboplatin, and gemcitabine in two patients with pure squamous cell carcinoma of the bladder and found that neither of the patients showed any response to chemotherapy [65]. 

Importantly, much of the clinical literature providing guidance on the treatment of SCC BC is based on urothelial carcinoma with squamous differentiation. This is an important distinction when considering the treatment of SCC BC, as it has been shown that therapeutics react differently depending on the histological percentage of squamous tissue effected [33].

## 4. Radiotherapy

The objective of RT in cancer treatment is to target the cancer at its source without causing destruction to the surrounding tissues. Previously, radiation has been shown to have a limited role in the treatment of SCC. Prempree et al. showed that, unless the SCC tumor is smaller than 3 cm with minimal muscle invasion, RT had little advantage [66]. Furthermore, Quilty et al. showed that the T3 local control rate of SCC was found to be 33.7% at 3 years and survival was only 18.3% at 3 years [67]. However, more recent advances in RT for BC may be able to provide targeted treatment at the source of the SCC in the wake of muscle invasion [68]. These advances have mainly focused on organ-sparing techniques that can deliver increased doses of radiation at the tumor location [68]. 

More recently, advances in dosage targeting, 3D conformational radiotherapy (3D-CRT), and intensity-modulated radiation therapy (IMRT) have been refined to afford patients increased quality of life and oncologic control [68]. 3D-CRT involves the use of computer-generated models of the patient’s specific tumor, so radiation can be delivered directly to the patient’s exact tumor location and avoid damaging the surrounding organs. Shen et al. retrospectively analyzed 109 patients with BC who received adjuvant pelvic 3D-CRT and found 1, 3, and 5-year OS rates of 80%, 48%, and 37%, respectively [69]. In a phase III clinical trial, it was found adjuvant 3D-CRT provided a significant increase in OS when compared to adjuvant chemotherapy for patients with locally invasive SCC of the bladder [70].

IMRT provides another personalized treatment method for BC that can avoid destruction of sensitive, functional tissues [71]. Like 3D-CRT, IMRT works by delivering personalized radiation doses to tumors using radiation beams that conform to the patient’s exact tumor. Hsieh et al. followed an elderly patient group treated with IMRT and found good locoregional control of the BC [72]. Of the 19 eligible elderly patients studied, 17 were found to have no local reoccurrence, which provides promise for the future of IMRT [72]. 

Volumetric-modulated arc therapy (VMAT) is a newer therapy that relies on the principle of IMRT. VMAT affords patients a targeted, tumor-specific therapy like IMRT, but enables even greater localization to the cancer site [73]. VMAT is optimized via variation of gantry speed multi-leaf positions, and dosage rate [73]. Foroudi et al. compared 3D-CRT to IMRT and VMAT and found similar tumor control and tissue complication probabilities amongst all three treatment modalities [74].

Though radiotherapy for BC is rapidly increasing in use, there is still a lack of evidence for the use of radiotherapy as a standardized treatment for SCC BC as few trials and studies have focused on this histological variant.

## 5. Targeted Immune Checkpoint Inhibitors

With RC being a considerably invasive procedure and SCC BC showing poor responsiveness to RT and chemotherapies, an increased amount of research is being invested in advancing therapies that target specific processes that inhibit cancer metastases. Very prevalent today is research in immune checkpoint inhibitors (ICI) as they have shown efficacy in traditional urothelial carcinoma settings. ICIs are quickly becoming the standard of care in many TCC BC settings, and may show promise for their use in SCC BC.

**Figure 2:**
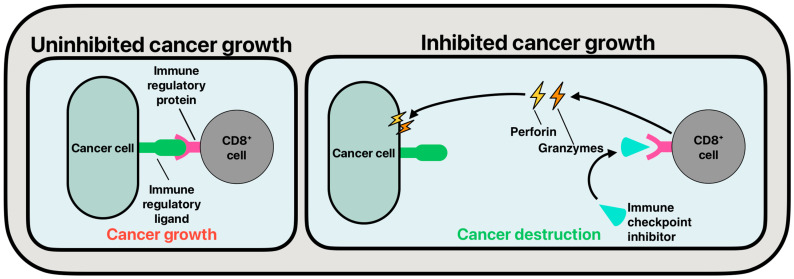
The common function of immune checkpoint inhibitors.

ICIs decrease the metastases of cancer by upregulating the immune response towards cancerous cells. ICIs work by decreasing the innate response the immune system has to normally healthy cells, where no cellular destruction will occur. In the case of cancerous cells, these neoplastic cells are capable of camouflaging themselves as normal cells, thus avoiding cellular destruction. Immune checkpoint proteins are prevalent on the surface of T-cells. When these checkpoint proteins are targeted by ICIs, which are monoclonal antibodies, their function is inhibited, allowing for a full immune response towards the cancerous cells present (Figure 2). When ICIs are active, CD8+ T-cells can release perforin and various granzymes to lyse cancerous cells in the body.

Programmed death ligand 1 (PD-L1) is a ligand to programmed death 1 (PD-1) and is an inhibitory factor to the immune response to cancer cells. Thus, when PD-L1 is treated with an ICI, cytotoxic T-cells are capable of lysing cancer cells. Very little clinical information is available regarding the efficacy of drugs that target and inhibit the PD-1/PD-L1 axis for SCC of the bladder due to the variant’s rarity and lack of incidence in developing nations. However, there is a growing wealth of knowledge regarding their use in the treatment of TCC of the bladder that is very promising. Pembrolizumab is an inhibitor of the PD-1 protein on the surface of T-cells. In an open label, phase III clinical trial for the treatment of advanced urothelial carcinoma, it was shown that a pembrolizumab treatment group gave an OS of 10.3 months versus 7.4 months for the chemotherapy treatment group [75].

Cytotoxic T lymphocyte-associated protein-4 (CTLA-4) is a protein expressed on the surface of T-cells that acts as protein that controls the bodies response to adverse cellular changes such as neoplasia. CTLA-4 can bind to co-stimulatory molecules on the surface of antigen-presenting cells, such as the B7 protein, that inhibit the destruction of cancerous cells by T-cells [76]. When CTLA-4/B7 binding is inhibited, the T-cell is free to enact cytotoxic activity on tumorigenic cells. CTLA-4 protein levels are upregulated in muscle-invasive bladder cancer and the CTLA-4-disrupted T-cells had greater cytotoxic anti-tumor effects [77]. In a murine model, it was found that a combination therapy with anti-PD-1 and anti-CTLA-4 antibodies gave a superior anti-tumor response than either treatment alone [78].

BRAF mutations, and more specifically the common BRAF V600 mutation, cause continuous activation of the MAPK/ERK pathway, which leads to continuous activation of tumor growth pathways. In cancers such as melanoma that are more prone to BRAF mutations, ICIs such as vemurafenib and dabrafenib target the pathway to decrease cancer spread [79]. BRAF mutations are not as common in bladder cancer as other types of cancers such as melanoma or colorectal cancer [80]. Though BRAF-mutated urothelial BC has not shown a decrease in overall survival, these mutations present an opportunity to pursue further clinical research in the application of combination therapy with both BRAF-targeting and PD-1/PD-L1 targeting ICIs [81]. Due to TCC being the most prevalent BC variant, there is a lack of research that aims to characterize SCC BC by biomarkers. This is an area that can be explored in the future, so patients can receive treatment that best suits their specific bladder cancer disease pathology.

Though ICIs show promise in the use of TCC, it is important to consider that SCC BC has been shown to react differently to other treatment modalities when compared to TCC. An important distinction when considering the use of ICIs for SCC BC is the lack of large-scale, randomized clinical trials for SCC variants specifically. Previous literature has already shown differing treatment reactions depending on the magnitude of squamous differentiation when treated with neoadjuvant chemotherapy. Before clinical trials for ICIs can be designed and implemented, clinicians need to characterize clear variables to define SCC BC by variation and presentation. Then, these groups can be treated based on their respective SCC BC disease classification.

## 6. Conclusions

Though the rates of SCC BC are decreasing in developing nations due to better control of schistosomiasis, there is still a high enough incidence of SCC in developing nations and in the western world that formal treatment guidelines are needed to attenuate the disease course of this rare histological variant. Efforts have been taken to decrease the rate of schistosomiases in endemic regions, that are sure to decrease the incidence of SCC BC in years to come. Various global health organizations have helped to decrease the price of the effective drug praziquantel that is used to treat schistosomiases. Further efforts are being made to raise awareness of schistosomiasis in developing nations. Though it still seems that most of the efforts being made for disease control are highly focused on decreasing morbidity, rather than decreasing transmission. As previous literature has shown, to control schistosomiasis infections, both proper transmission control and disease control will have to be implemented congruently to stop the causative parasite completely. The endemic regions of the world still have further progress to make before complete awareness and decreased transmission of schistosomiasis can occur.

RC remains the standard for MIBC and the standard for SCC BC. With SCC typically presenting in invasive stages, RC is suspected to be the standard treatment for years to come until other less invasive therapeutics can achieve better disease control. The drawback of RC is that it can be destructive to surrounding tissues, is generally invasive, and relies on the lack of error of the surgeon. Further, because SCC is usually found in its advanced stages with an increased chance of metastases, patients will usually be candidates for lymph node resection, which is also invasive. SCC BC is complex due to its lack of response to traditional neoadjuvant and adjuvant treatment methods such as RT and chemotherapy, though in recent years these therapeutic methods are gaining increased refinement and technology and may someday be as effective as RC for SCC BC.

It is evident that there is an increased need for more targeted therapeutics and early prognostic indicators of SCC BC that will attenuate its spread to muscle-invasive stages. Recent research looking at immune-regulatory proteins such as PD-1, PD-L1, and CTLA-4 may show promise as early indicators of SCC disease spread. These immune regulatory proteins may also present as possible drug targets to enhance the destruction of SCC BC, as seen in the growing body of research using ICIs for TCC BC, where ICIs are becoming the standard of care in some cases. Though more research into these ICIs is needed to discern the potential efficacy of these drugs for SCC BC specifically. There is a need for more research and clinical trials for specific SCC BC indicators and treatments that will benefit patients with this rare histological variant, that differs drastically in disease course from typical BC variants.

Overall, it is apparent that formal treatment guidelines need to be set in place for SCC BC pathologies. Many of the studies evaluating possible treatment modalities for SCC BC involve retrospective analyses of diverse treatment populations with a lack of similar treatment variables. Further, the attempts at classifying possible treatment guidelines and modalities seem to be delayed as many have not considered that the disease’s progression and treatment seem to heavily depend on the amount of squamous variation present, whether urothelial with squamous differentiation or a purely squamous variant. Before clinical guidelines and regulations can be made, a thorough understanding of this disease will need to be had.

To better ensure effective treatment of SCC BC in the future, clinicians are going to need to devise a solid system that defines the amount of squamous variation present in the tissues. There is a need for more research and large-scale, randomized clinical trials for specific SCC BC indicators and treatments that will benefit patients with this rare histological variant, that differs in disease course from typical BC variants.

## Figures and Tables

**Table 1:** TNM staging system for bladder cancer.

**Tumor (T)—Size of the tumor**	
Ta	Cancer found only in the innermost layer of the bladder lining
T1	Cancer has started to grow into the connective tissue beneath the bladder lining
T2	Cancer has grown through the connective tissue into the muscle
T2a	Cancer has grown into the superficial muscle
T2b	Cancer has grown into the deeper muscle
T3	Cancer has grown through the muscle into the fat layer
T3a	Cancer in the fat layer can only be seen under a microscope
T3b	Cancer in the fat layer can be seen on testes or felt by your doctor
T4	Cancer has spread outside the bladder
T4a	Cancer has spread to the prostate, womb (uterus), or vagina
T4b	Cancer has spread to the wall of the pelvis or abdomen
**Node (N)—Lymph node spread**	
N0	No cancer cells in any lymph nodes
N1	Cancer cells in one lymph node in the pelvis
N2	Cancer cells in more than one lymph node in the pelvis
N3	Cancer cells in one or more lymph node just outside the pelvis
**Metastasis (M)—Metastatic spread around the body**	
M0	Cancer has not spread to other parts of the body
M1	Cancer has spread to other parts of the body
M1a	Cancer has spread to the lymph nodes outside the pelvis
M1b	Cancer has spread to other parts of the body like the bones, lungs, or liver

**Table 2:** Outcomes for treatments involving squamous cell carcinoma of the bladder with chemotherapeutics.

Authors	Treatment Modality	Clinical Setting	Treatment Setting	Outcome(s)
Khaled, H.M., et al. [56]	Neoadjuvant gemcitabine and cisplatin-based chemotherapy.	Patients with newly diagnosed bladder cancer (T3–4, N0–2, M0). This was a heterogeneous study that included patients with transitional cell carcinoma and squamous cell carcinoma.	Patients were randomized to radical cystectomy alone or initial 3 cycles of gemcitabine/cisplatin, then managed according to response.	The overall response rate to gemcitabine/cisplatin was 55.1%, and complete response was achieved in 28.6%. The 3-year overall survival was 51.9% versus 51.2% in the chemotherapy and surgery arms, respectively. The 3-year disease-free survival was 31.8% in the chemotherapy treatment group.
Speir, R.W., et al. [33]	Cisplatin-based neoadjuvant chemotherapy.	Patients with urothelial bladder cancer presenting with a component of squamous variant histology at the time of transurethral resection.	Patients with squamous variant bladder cancer who either underwent neoadjuvant cisplatin-based chemotherapy or no neoadjuvant chemotherapy prior to radical cystectomy.	The overall survival and recurrence-free survival were clinically greater in the bladder cancer groups with <50% squamous variation when treated with neoadjuvant chemotherapy.
Minato, A., et al. [61]	Cisplatin-based neoadjuvant chemotherapy.	Patients with invasive bladder cancer (clinical T3–4aN0M0) who presented with urothelial carcinoma with squamous differentiation.	Patients were treated with 2 or 3 cycles of neoadjuvant chemotherapy followed by radical cystectomy.	Pathologic complete response without residual tumors was not seen in any patients with urothelial carcinoma with squamous differentiation.
Dotson, A., et al. [62]	Cisplatin-based neoadjuvant chemotherapy.	Patients with muscle-invasive squamous cell carcinoma who had underwent radical cystectomy.	Patients were treated with radical cystectomy alone or neoadjuvant chemotherapy prior to radical cystectomy.	Neoadjuvant chemotherapy did not confer survival advantage. The overall survival was equivalent between those who received neoadjuvant chemotherapy and did not receive neoadjuvant chemotherapy.
Kastritis, E., et al. [63]	Carboplatin or cisplatin-based chemotherapy.	Advanced or metastatic urothelial cancer that is purely squamous cell histology.	Patients received frontline platinum (cisplatin or carboplatin)-based chemotherapy.	Response rate was 27% for patients with squamous cell carcinoma with a median survival of 13.6 months.

## Abbreviations

3D-CRT	3D conformational radiotherapy
BC	Bladder Cancer
CSS	Cancer survival rate
CTLA-4	Cytotoxic T lymphocyte-associated protein-4
ICI	Immune checkpoint inhibitors
IMRT	Intensity modulated radiation therapy
LN	Lymph node
MIBC	Muscle-invasive bladder cancer
OS	Overall survival
PD-1	Programmed death 1
PD-L1	Programmed death ligand 1
RC	Radical Cystectomy
SCC	Squamous Cell Carcinoma
TCC	Transitional Cell Carcinoma
TURBT	transurethral resection of the bladder tumor
UC	Urothelial Carcinoma
VMAT	Volumetric-modulated arc therapy

## References

[1] Bray F., Laversanne M., Sung H., Ferlay J., Siegel R.L., Soerjomataram I., Jemal A. (2024). Global cancer statistics 2022: GLOBOCAN estimates of incidence and mortality worldwide for 36 cancers in 185 countries. CA Cancer J. Clin..

[2] Zhang Y., Rumgay H., Li M., Yu H., Pan H., Ni J. (2023). The global landscape of bladder cancer incidence and mortality in 2020 and projections to 2040. J. Glob. Health.

[3] Jubber I., Ong S., Bukavina L., Black P.C., Compérat E., Kamat A.M., Kiemeney L., Lawrentschuk N., Lerner S.P., Meeks J.J. (2023). Epidemiology of bladder cancer in 2023: A systematic review of risk factors. Eur. Urol..

[4] Lobo N., Shariat S.F., Guo C.C., Fernandez M.I., Kassouf W., Choudhury A., Gao J., Williams S.B., Galsky M.D., Taylor J.A. (2020). What Is the Significance of Variant Histology in Urothelial Carcinoma?. Eur. Urol. Focus..

[5] Deuker M., Martin T., Stolzenbach F., Rosiello G., Collà Ruvolo C., Nocera L., Tian Z., Becker A., Kluth L., Roos F.C. (2021). Bladder Cancer: A Comparison Between Non-urothelial Variant Histology and Urothelial Carcinoma Across All Stages and Treatment Modalities. Clin. Genitourin. Cancer.

[6] Johnson D., Schoenwald M., Ayala A., Miller L. (1976). Squamous cell carcinoma of the bladder. J. Urol..

[7] Lagwinski N., Thomas A., Stephenson A.J., Campbell S., Hoschar A.P., El-Gabry E., Dreicer R., Hansel D.E. (2007). Squamous Cell Carcinoma of the Bladder: A Clinicopathologic Analysis of 45 Cases. Am. J. Surg. Pathol..

[8] Costello A.J., Tiptaft R.C., England H.R., Blandy J.E. (1984). Squamous cell carcinoma of bladder. Urology.

[9] Kassouf W., Spiess P.E., Siefker-Radtke A., Swanson D., Grossman H.B., Kamat A.M., Munsell M.F., Guo C.C., Czerniak B.A., Dinney C.P. (2007). Outcome and patterns of recurrence of nonbilharzial pure squamous cell carcinoma of the bladder: A contemporary review of The University of Texas M D Anderson Cancer Center experience. Cancer.

[10] El-Sebaie M., Zaghloul M., Howard G., Mokhtar A. (2005). Squamous cell carcinoma of the bilharzial and non-bilharzial urinary bladder: A review of etiological features, natural history, and management. Int. J. Clin. Oncol..

[11] Berry A., Iriart X., Fillaux J., Magnaval J.F. (2017). Schistosomose urogénitale et cancer. Bull. Société Pathol. Exot..

[12] Sung H., Ferlay J., Siegel R.L., Laversanne M., Soerjomataram I., Jemal A., Bray F. (2021). Global Cancer Statistics 2020: GLOBOCAN Estimates of Incidence and Mortality Worldwide for 36 Cancers in 185 Countries. CA Cancer J. Clin..

[13] Martin J.W., Carballido E.M., Ahmed A., Farhan B., Dutta R., Smith C., Youssef R.F. (2016). Squamous cell carcinoma of the urinary bladder: Systematic review of clinical characteristics and therapeutic approaches. Arab. J. Urol..

[14] Ng K. (2022). The Etiology of Bladder Cancer.

[15] Rambau P.F., Chalya P.L., Jackson K. (2013). Schistosomiasis and urinary bladder cancer in North Western Tanzania: A retrospective review of 185 patients. Infect. Agents Cancer.

[16] Bowa K., Mulele C., Kachimba J., Manda E., Mapulanga V., Mukosai S. (2018). A review of bladder cancer in Sub-Saharan Africa: A different disease, with a distinct presentation, assessment, and treatment. Ann. Afr. Med..

[17] Efared B., Bako A., Idrissa B., Alhousseini D., Boureima H., Sodé H., Nouhou H. (2022). Urinary bladder Schistosoma haematobium-related squamous cell carcinoma: A report of two fatal cases and literature review. Trop. Dis. Travel. Med. Vaccines.

[18] Khalaf I., Shokeir A., Shalaby M. (2012). Urologic complications of genitourinary schistosomiasis. World J. Urol..

[19] Ghoneim M.A. (2002). Bilharziasis of the genitourinary tract. BJU Int..

[20] Rausch S., Lotan Y., Youssef R.F. (2014). Squamous cell carcinogenesis and squamous cell carcinoma of the urinary bladder: A contemporary review with focus on nonbilharzial squamous cell carcinoma. Urol. Oncol. Semin. Orig. Investig..

[21] El-Bolkainy M., Mokhtar N., Ghoneim M., Hussein M. (1981). The impact of schistosomiasis on the pathology of bladder carcinoma. Cancer.

[22] Khalaf I., El-Mallah E., Elsotouhi I., Abu-Zeid H., Elmeligy A. (2008). Pathologic pattern of invasive bladder carcinoma: Impact of bilharziasis. Afr. J. Urol..

[23] Magaisa K., Taylor M., Kjetland E.F., Naidoo P.J. (2015). A review of the control of schistosomiasis in South Africa. South. Afr. J. Sci..

[24] Gouda I., Mokhtar N., Bilal D., El-Bolkainy T., El-Bolkainy N.M. (2007). Bilharziasis and bladder cancer: A time trend analysis of 9843 patients. J. Egypt. Natl. Cancer Inst..

[25] Sokolow S., Wood C., Jones I., Swartz S., Lopez M., Hsieh M., Lafferty K., Kuris A., Rickards C., De Leo G. (2016). Global Assessment of Schistosomiasis Control Over the Past Century Shows Targeting the Snail Intermediate Host Works Best. PLoS Neglected Trop. Dis..

[26] Sarant L. (2017). Egypt: The flatworm’s revenge. Nature.

[27] Evan Secor W. (2014). Water-based interventions for schistosomiasis control. Pathog. Glob. Health.

[28] King C., Bertsch D. (2015). Historical Perspective: Snail Control to Prevent Schistosomiasis. PLoS Neglected Trop. Dis..

[29] El-Kassas M., Sheemy R.E., Elbadry M. (2024). Strategies and achievements in controlling and eliminating schistosomiasis from Egypt. Egypt. Liver J..

[30] Southgate V.R., Rollinson D., Tchuenté L.A.T., Hagan P. (2024). Towards control of schistosomiasis in sub-Saharan Africa. J. Helminthol..

[31] World Health Organization (2013). Regional Strategic Plan for Neglected Tropical Diseases in the African Region 2014–2020.

[32] Aula O., McManus D., Jones M., Gordon C. (2021). Schistosomiasis with a Focus on Africa. Trop. Med. Infect. Dis..

[33] Speir R.W., Barboza M.P., Calaway A., Masterson T.A., Cary C., Koch M., Bihrle R., Cheng L., Adra N., Kaimakliotis H. (2021). Role of Neoadjuvant Chemotherapy in Squamous Variant Histology in Urothelial Bladder Cancer: Does Presence and Percentage Matter?. Clin. Genitourin. Cancer.

[34] Lonati C., Baumeister P., Ornaghi P., Di Trapani E., De Cobelli O., Rink M., Karnes R., Poyet C., Simone G., Afferi L. (2022). Accuracy of Transurethral Resection of the Bladder in Detecting Variant Histology of Bladder Cancer Compared with Radical Cystectomy. Eur. Urol. Focus..

[35] Celis J.E., Rasmussen H.H., Vorum H., Madsen P., Honoré B., Wolf H., Orntoft T.F. (1996). Bladder squamous cell carcinomas express psoriasin and externalize it to the urine. J. Urol..

[36] Badr K., Nolen J.D.L., Derose P., Cohen C. (2004). Muscle invasive schistosomal squamous cell carcinoma of the urinary bladder: Frequency and prognostic significance of p53, BCL-2, HER2/neu, and proliferation (MIB-1). Hum. Pathol..

[37] Chaudhary K.S., Lu Q.L., Abel P.D., Khandan Nia N., Shoma A.M., El Baz M.A., Stamp G.W.H., Lalani E.N. (2003). Expression of bcl-2 and p53 oncoproteins in schistosomiasis-associated transitional and squamous cell carcinoma of urinary bladder. Br. J. Urol..

[38] Rundle J.S., Hart A.J., McGeorge A., Smith J.S., Malcolm A.J., Smith P.M. (1982). Squamous cell carcinoma of bladder. A review of 114 patients. Br. J. Urol..

[39] Serretta V., Pomara G., Piazza F., Gange E. (2000). Pure squamous cell carcinoma of the bladder in western countries. Report on 19 consecutive cases. Eur. Urol..

[40] Navon J.D., Soliman H., Khonsari F., Ahlering T. (1997). Screening cystoscopy and survival of spinal cord injured patients with squamous cell cancer of the bladder. J. Urol..

[41] Stonehill W.H., Goldman H.B., Dmochowski R.R. (1997). The use of urine cytology for diagnosing bladder cancer in spinal cord injured patients. J. Urol..

[42] Shokeir A.A. (2004). Squamous cell carcinoma of the bladder: Pathology, diagnosis and treatment. BJU Int..

[43] Manley K.V., Hubbard R., Swallow D., Finch W., Wood S.J., Biers S.M. (2017). Risk factors for development of primary bladder squamous cell carcinoma. Ann. R. Coll. Surg. Engl..

[44] Chavan S., Bray F., Lortet-Tieulent J., Goodman M., Jemal A. (2014). International Variations in Bladder Cancer Incidence and Mortality. Eur. Urol..

[45] Parkin D.M. (2010). The global burden of urinary bladder cancer. Scand. J. Urol. Nephrol..

[46] Sathianathen N.J., Kalapara A., Frydenberg M., Lawrentschuk N., Weight C.J., Parekh D., Konety B.R. (2019). Robotic Assisted Radical Cystectomy vs Open Radical Cystectomy: Systematic Review and Meta-Analysis. J. Urol..

[47] Tang K., Li H., Xia D., Hu Z., Zhuang Q., Liu J., Xu H., Ye Z. (2014). Laparoscopic versus Open Radical Cystectomy in Bladder Cancer: A Systematic Review and Meta-Analysis of Comparative Studies. PLoS ONE.

[48] Deuker M., Franziska Stolzenbach L., Rosiello G., Luzzago S., Martin T., Tian Z., Tilki D., Shariat S.F., Saad F., Kassouf W. (2021). Radical cystectomy improves survival in patients with stage T1 squamous cell carcinoma and neuroendocrine carcinoma of the urinary bladder. Eur. J. Surg. Oncol..

[49] Abdel-Rahman O. (2017). Squamous cell carcinoma of the bladder: A SEER database analysis. Clin. Genitourin. Cancer.

[50] Debbagh A., Bennani S., Hafiani M., el Mrini M., Benjelloun S. (1997). Epidermoid carcinoma of the bladder. Apropos of 14 cases. Ann. Urol..

[51] Kiss B., Burkhard F.C., Thalmann G.N. (2016). Open radical cystectomy: Still the gold standard for muscle invasive bladder cancer. World J. Urol..

[52] Chang S.S., Hassan J.M., Cookson M.S., Wells N., Smith J.A. (2003). Delaying Radical Cystectomy for Muscle Invasive Bladder Cancer Results in Worse Pathological Stage. J. Urol..

[53] Chu A.T., Holt S.K., Wright J.L., Ramos J.D., Grivas P., Yu E.Y., Gore J.L. (2019). Delays in radical cystectomy for muscle-invasive bladder cancer. Cancer.

[54] Boeri L., Soligo M., Frank I., Boorjian S.A., Thompson R.H., Tollefson M., Quevedo F.J., Cheville J.C., Karnes R.J. (2019). Delaying Radical Cystectomy After Neoadjuvant Chemotherapy for Muscle-invasive Bladder Cancer is Associated with Adverse Survival Outcomes. Eur. Urol. Oncol..

[55] Bandini M., Pederzoli F., Madison R., Briganti A., Ross J.S., Niegisch G., Yu E.Y., Bamias A., Agarwal N., Sridhar S.S. (2020). Unfavorable Cancer-specific Survival After Neoadjuvant Chemotherapy and Radical Cystectomy in Patients With Bladder Cancer and Squamous Cell Variant: A Multi-institutional Study. Clin. Genitourin. Cancer.

[56] Khaled H.M., Shafik H.E., Zabhloul M.S., Ghoneim M., Saber R.A., Manie M., Enein H.A., Megeed H.A., Mansur O., Sherbini M.E. (2014). Gemcitabine and Cisplatin as Neoadjuvant Chemotherapy for Invasive Transitional and Squamous Cell Carcinoma of the Bladder: Effect on Survival and Bladder Preservation. Clin. Genitourin. Cancer.

[57] Matulay J.T., Woldu S.L., Lim A., Narayan V.M., Li G., Kamat A.M., Anderson C.B. (2019). The impact of squamous histology on survival in patients with muscle-invasive bladder cancer. Urol. Oncol. Semin. Orig. Investig..

[58] Galsky M.D., Iasonos A., Mironov S., Scattergood J., Donat S.M., Bochner B.H., Herr H.W., Russo P., Boyle M.G., Bajorin D.F. (2007). Prospective trial of ifosfamide, paclitaxel, and cisplatin in patients with advanced non-transitional cell carcinoma of the urothelial tract. Urology.

[59] Zaghloul M.S., Christodouleas J.P., Smith A., Abdalla A., William H., Khaled H.M., Hwang W.-T., Baumann B.C. (2016). A randomized clinical trial comparing adjuvant radiation versus chemo-RT versus chemotherapy alone after radical cystectomy for locally advanced bladder cancer. J. Clin. Oncol..

[60] Spiess P.E., Agarwal N., Bangs R., Boorjian S.A., Buyyounouski M.K., Clark P.E., Downs T.M., Efstathiou J.A., Flaig T.W., Friedlander T. (2017). Bladder Cancer, Version 5.2017, NCCN Clinical Practice Guidelines in Oncology. J. Natl. Compr. Cancer Netw..

[61] Minato A., Fujimoto N., Kubo T. (2017). Squamous Differentiation Predicts Poor Response to Cisplatin-Based Chemotherapy and Unfavorable Prognosis in Urothelial Carcinoma of the Urinary Bladder. Clin. Genitourin. Cancer.

[62] Dotson A., May A., Davaro F., Raza S.J., Siddiqui S., Hamilton Z. (2019). Squamous cell carcinoma of the bladder: Poor response to neoadjuvant chemotherapy. Int. J. Clin. Oncol..

[63] Kastritis E., Dimopoulos M.-A., Antoniou N., Deliveliotis C., Chrisofos M., Skolarikos A., Gika D., Bamias A. (2006). The Outcome of Patients with Advanced Pure Squamous or Mixed Squamous and Transitional Urothelial Carcinomas Following Platinum-based Chemotherapy. Anticancer. Res..

[64] Miyata Y., Watanabe S.-I., Takahashi H., Sagara Y., Matsuo T., Ohba K., Sakai H. (2011). Effect of Maintenance Chemotherapy with Gemcitabine and Paclitaxel on Recurrent Squamous Cell Carcinoma of the Bladder: A Case Report. Anticancer. Res..

[65] Hussain M., Vaishampayan U., Du W., Redman B., Smith D.C. (2001). Combination Paclitaxel, Carboplatin, and Gemcitabine Is an Active Treatment for Advanced Urothelial Cancer. J. Clin. Oncol..

[66] Prempree T., Amornmarn R. (1984). Radiation Management of Squamous Cell Carcinoma of the Bladder. Acta Radiol. Oncol..

[67] Quilty P.M., Duncan W. (1986). Radiotherapy for squamous carcinoma of the urinary bladder. Int. J. Radiat. Oncol. Biol. Phys..

[68] Zhang S., Yu Y.H., Zhang Y., Qu W., Li J. (2015). Radiotherapy in muscle-invasive bladder cancer: The latest research progress and clinical application. Am. J. Cancer Res..

[69] Shen J., Liu X., Zhang F., Hu K., Hou X., Lian X., Sun S. (2009). Outcome of three-dimensional conformal radiotherapy for 109 patients with bladder cancer. Chin. J. Radiat. Oncol..

[70] Zaghloul M.S., Christodouleas J.P., Zaghloul T., Abdalla A., William H., Khaled H.M., Hwang W.-T., Baumann B.C. (2020). Prospective trial of adjuvant therapy after radical cystectomy for locally advanced bladder cancer: Analysis of the squamous cell carcinoma subgroup. J. Clin. Oncol..

[71] Sanghani M., Mignano J. (2006). Intensity Modulated Radiation Therapy: A Review of Current Practice and Future Directions. Technol. Cancer Res. Treat..

[72] Hsieh C.H., Chung S.D., Chan P.H., Lai S.K., Chang H.C., Hsiao C.H., Wu L.J., Chong N.S., Chen Y.J., Wang L.Y. (2011). Intensity modulated radiotherapy for elderly bladder cancer patients. Radiat. Oncol..

[73] Guckenberger M., Richter A., Krieger T., Wilbert J., Baier K., Flentje M. (2009). Is a single arc sufficient in volumetric-modulated arc therapy (VMAT) for complex-shaped target volumes?. Radiother. Oncol..

[74] Foroudi F., Wilson L., Bressel M., Haworth A., Hornby C., Pham D., Cramb J., Gill S., Tai K.H., Kron T. (2012). A dosimetric comparison of 3D conformal vs intensity modulated vs volumetric arc radiation therapy for muscle invasive bladder cancer. Radiat. Oncol..

[75] Bellmunt J., de Wit R., Vaughn David J., Fradet Y., Lee J.-L., Fong L., Vogelzang Nicholas J., Climent Miguel A., Petrylak Daniel P., Choueiri Toni K. (2017). Pembrolizumab as Second-Line Therapy for Advanced Urothelial Carcinoma. N. Engl. J. Med..

[76] Sobhani N., Tardiel-Cyril D.R., Davtyan A., Generali D., Roudi R., Li Y. (2021). CTLA-4 in Regulatory T Cells for Cancer Immunotherapy. Cancers.

[77] Zhang W., Long S., Zhao Z., Du P., Ye X., Li D., Cai Z., Han J., Cai J. (2019). Disruption of CTLA-4 expression on peripheral blood CD8 + T cell enhances anti-tumor efficacy in bladder cancer. Cancer Chemother. Pharmacol..

[78] van Hooren L., Sandin L.C., Moskalev I., Ellmark P., Dimberg A., Black P., Tötterman T.H., Mangsbo S.M. (2017). Local checkpoint inhibition of CTLA-4 as a monotherapy or in combination with anti-PD1 prevents the growth of murine bladder cancer. Eur. J. Immunol..

[79] Luke J., Hodi F.S. (2013). Ipilimumab, Vemurafenib, Dabrafenib, and Trametinib: Synergistic Competitors in the Clinical Management of BRAF Mutant Malignant Melanoma. Oncologist.

[80] Boulalas I., Zaravinos A., Delakas D., Spandidos D.A. (2009). Mutational analysis of the BRAF gene in transitional cell carcinoma of the bladder. Int. J. Biol. Markers.

[81] Grivas P., Lalani A.-K.A., Pond G.R., Nagy R.J., Faltas B., Agarwal N., Gupta S.V., Drakaki A., Vaishampayan U.N., Wang J. (2020). Circulating tumor DNA alterations in advanced urothelial carcinoma and association with clinical outcomes: A pilot study. Eur. Urol. Oncol..

